# Pathological Study of a Case of Catastrophic Antiphospholipid Syndrome in a Patient With Gastrointestinal Bleeding

**DOI:** 10.7759/cureus.82263

**Published:** 2025-04-14

**Authors:** Brian Shih, Qi Zhang, Hao Li

**Affiliations:** 1 Medical School, Schulich School of Medicine, London, CAN; 2 Pathology and Laboratory Medicine, London Health Sciences Centre, London, CAN; 3 Clinical Neurosciences, Schulich School of Medicine, London, CAN

**Keywords:** anticoagulation, antiphospholipid syndrome, catastrophic antiphospholipid syndrome, gastrointestinal bleed, myocardial infarction, thrombosis

## Abstract

Catastrophic antiphospholipid syndrome (CAPS) is a very rare, life-threatening form of antiphospholipid syndrome (APS) characterized by multiorgan vascular occlusive events that occur rapidly in succession or simultaneously. Here, we describe a rare case of CAPS in a 67-year-old woman with a 26-year history of APS managed on therapeutic doses of warfarin presenting to hospital with a four-day history of melena secondary to gastrointestinal bleed. She was treated endoscopically, provided vitamin K for warfarin reversal, and started on dalteparin daily. During her course in hospital, she developed dyspnea and hypoxia with bilateral pleural effusions. The patient concurrently became increasingly hemodynamically unstable. She was treated with furosemide and broad-spectrum antibiotics but deteriorated, experienced cardiac arrest, and could not be successfully resuscitated. On autopsy, widespread microthrombi in cardiac, pulmonary, and cerebral tissues were identified and a diagnosis of CAPS was made. This case highlights the difficulty and importance of recognizing clinical manifestations of CAPS and balancing the risk for thrombosis when patients are undergoing treatment for concurrent serious health issues.

## Introduction

Antiphospholipid syndrome (APS) is an autoimmune condition characterized by thrombosis associated with circulating antiphospholipid antibodies affecting approximately five in 100,000 people [1]. Catastrophic antiphospholipid syndrome (CAPS) is a very rare and severe variant of APS affecting approximately 1% of APS patients that is associated with multiorgan thrombotic events that occur simultaneously or in succession and has a mortality rate of approximately 30-50%. Treatment requires a timely diagnosis and aggressive administration of anticoagulants, corticosteroids, and plasma exchange. However, diagnostic clarity can be poor given the rapid progression and non-specific symptoms/signs associated with this condition. Appropriate management is further complicated when patients present with concurrent life-threatening conditions such as bleeding. In this report, we present a 67-year-old woman with a 26-year history of APS with lupus anticoagulant (LA) managed on therapeutic doses of warfarin who initially presented to hospital with anemia in the context of gastrointestinal bleed. She experienced an episode of CAPS during admission, deteriorated rapidly, and could not be successfully resuscitated, with a diagnosis of CAPS made on autopsy based on histological evidence of multisystem thrombosis. This case highlights the difficulty in diagnosing, preventing, and managing CAPS when balancing it against another serious medical condition.

## Case presentation

**Figure 1:**
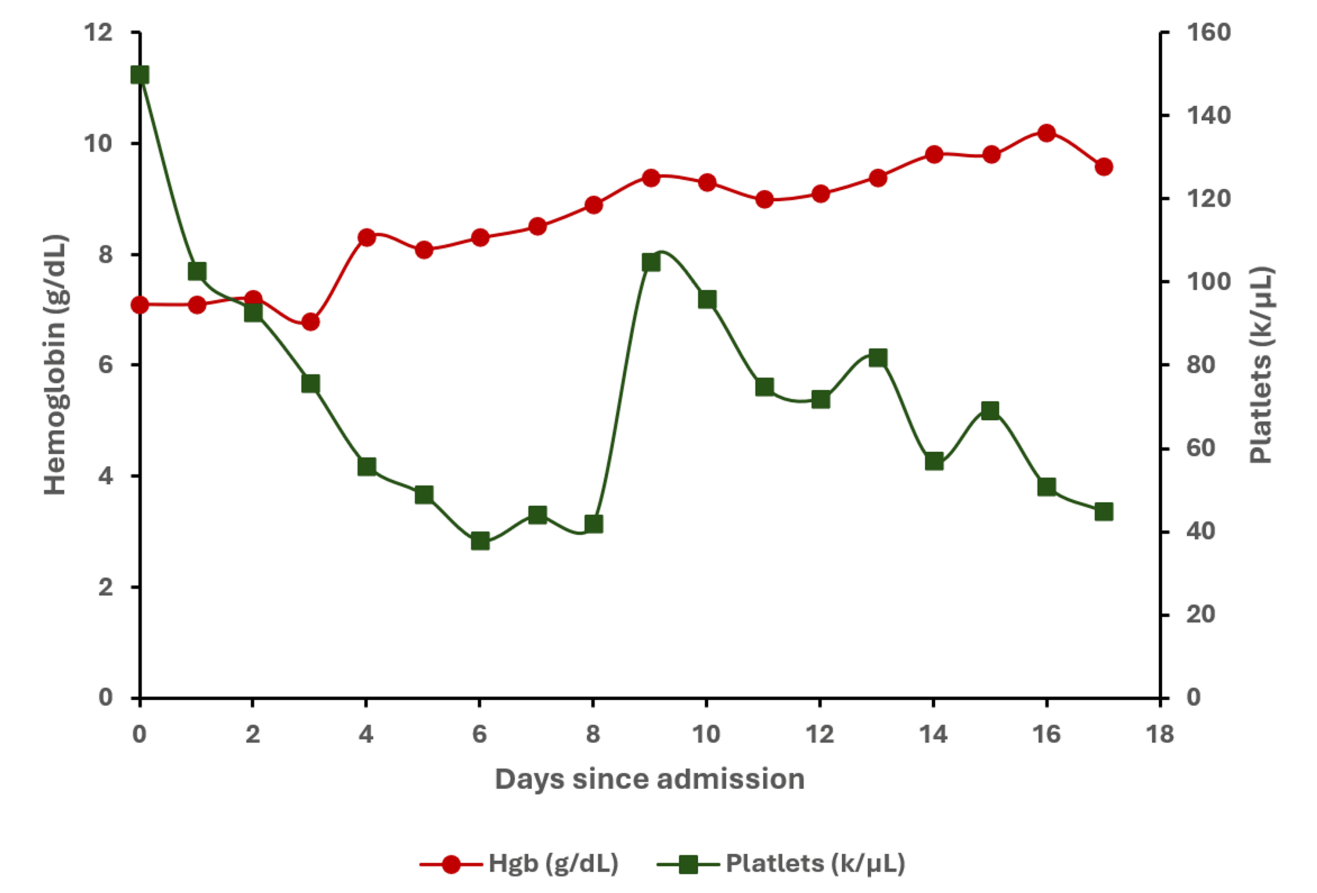
Trend of hemoglobin (g/dL) and platelets (k/µL) during the course of admission to death. Packed RBC administered on Day 0 and Day 3. Platelet transfusion administered on Day 8.

**Figure 2:**
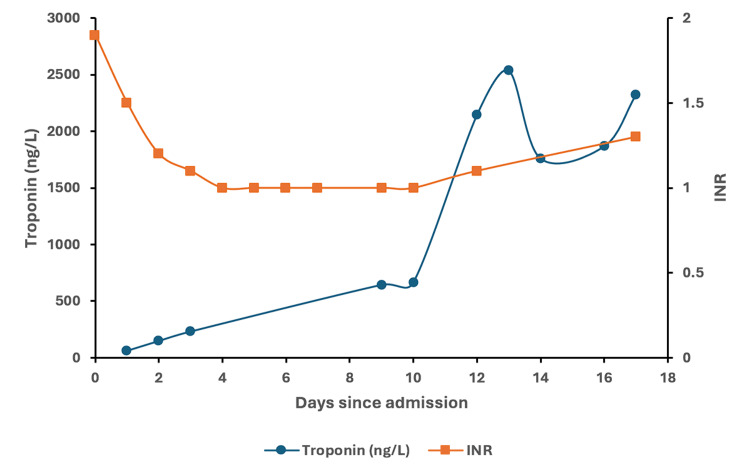
Trend of troponin (ng/L) and international normalized ratio (INR) during the course of admission to death.

**Figure 3:**
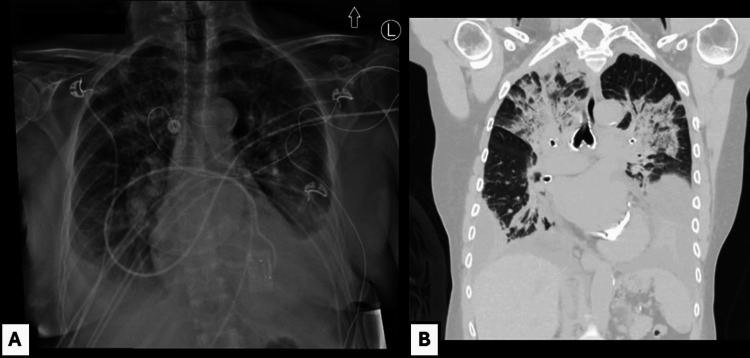
Thoracic radiology. (A) Chest X-Ray, anteroposterior, performed nine days after admission demonstrating bilateral pleural effusions. (B) Computed tomography (CT) of the chest (lung window) performed 11 days after admission showing bilateral upper lobe consolidation.

A 67-year-old woman with a history of mixed aortic stenosis-regurgitation, APS with LA for 26 years managed on warfarin with international normalized ratio (INR) monitoring, pulmonary embolism, deep vein thrombosis (DVT), multiple transient ischemic attacks, and recurrent pregnancy losses over 10 weeks gestation, presented with a four-day history of melena stools and was admitted. She was previously anti-cardiolipin and anti-beta2 glycoprotein-1 antibody negative. On admission, she had a hemoglobin of 6.7 g/dL, platelet count of 150 k/µL, INR of 3.0, partial thromboplastin time (PTT) of 51 secs, fibrinogen of 2.35 g/L, white blood cell count of 7.7 k/µL, creatinine of 1.13 mg/dL (at baseline) and total bilirubin of 5.5 μmol/L (Figure 1). She was transfused with 1U of packed red blood cells (pRBC) and given vitamin K for warfarin reversal which brought her INR down to 1.9. Her warfarin was replaced with 5000 IU of subcutaneous dalteparin daily which was continued throughout the course of admission for DVT prophylaxis. An esophagogastroduodenoscopy (EGD) revealed the source of bleeding to be in the duodenum which was treated endoscopically. Following the procedure, she developed epigastric pain and her troponin rose (Figure 2). The patient denied chest pain, shortness of breath, palpitations, and syncope. Initial clinical suspicion was type II myocardial infarction secondary to upper gastrointestinal (GI) bleed. Following this rise in troponin, an electrocardiograph (EKG) found no changes and an echocardiogram showed no significant acute findings with no wall motion abnormalities and normal ejection fraction. Her hemoglobin and platelet count continued to decrease following her initial EGD (Figure 1) and she was transfused with another 1U of pRBC and 1U of platelets. She developed an acute kidney injury (AKI) with a creatinine of 2.43 mg/dL and was treated for prerenal AKI with IV fluids. She had a repeat EGD that identified no active bleeding and a colonoscopy that was unremarkable for a lower GI bleed. Nine days following admission, she developed dyspnea with hypoxia. A chest X-ray revealed bilateral pleural effusions (Figure 3A). Further computed tomography (CT) demonstrated bilateral upper lobe consolidation (Figure 3B). At this point in time the differential diagnosis included pulmonary edema from congestive heart failure exacerbation, superimposed hospital-acquired pneumonia, pulmonary embolism, and interstitial lung disease. She was treated with one dose of 2 g ceftriaxone and a five-day course of 4.5 g piperacillin-tazobactam. Blood cultures and respiratory cultures were negative. She remained on her daily dalteparin during hospitalization and was not restarted on warfarin. A computed tomography pulmonary angiogram (CTPA) was not performed due to her persistently elevated creatinine. Concurrently, she developed supraventricular tachycardia (SVT) and became increasingly hemodynamically unstable with her troponin levels continuing to rise since admission to the 2000s (Figure 2). She deteriorated, experienced cardiac arrest, and could not be successfully resuscitated.

**Figure 4:**
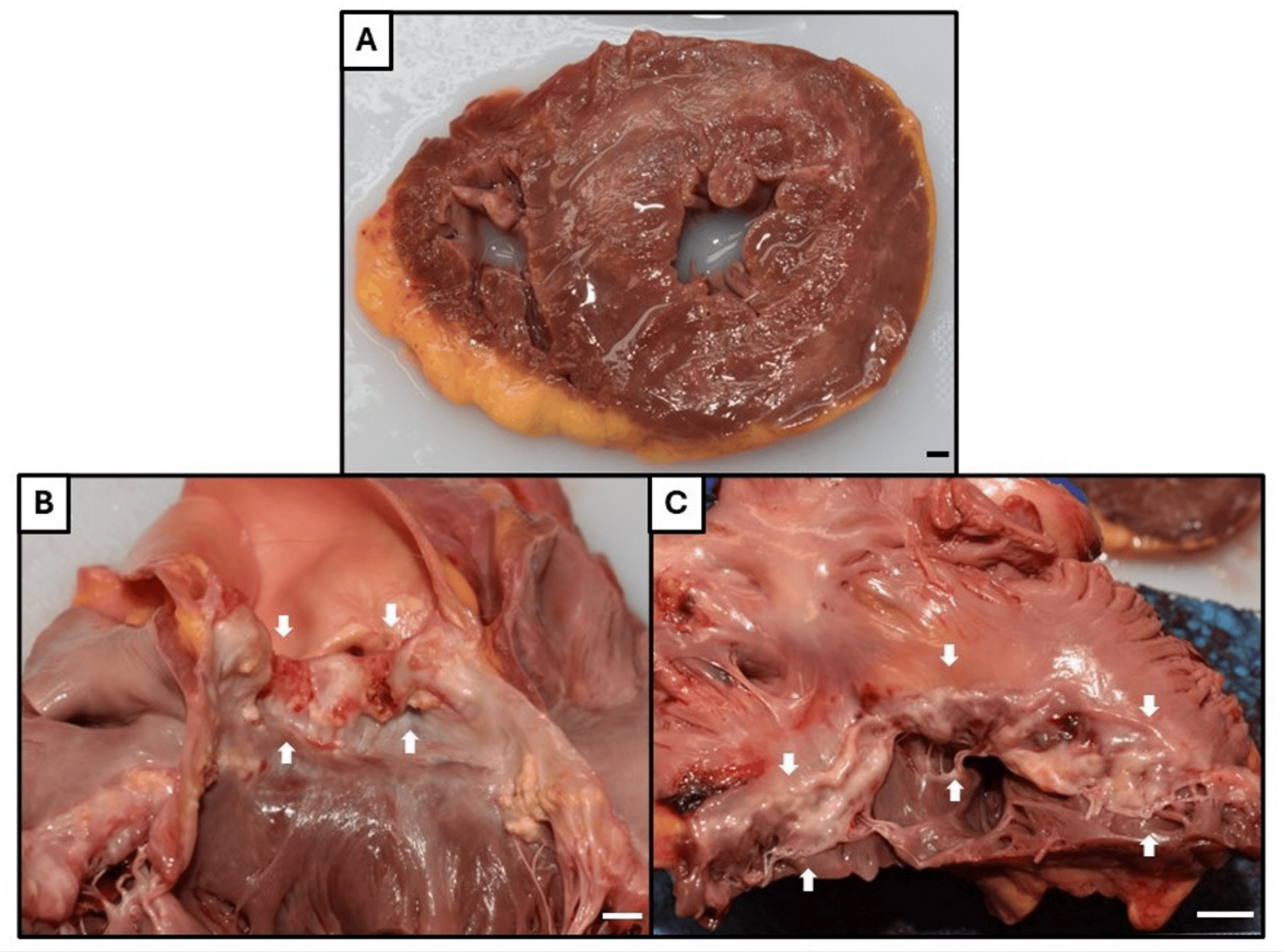
Postmortem macroscopic examination of the heart. (A) Cross section through ventricles (left ventricle on right, right ventricle on left, according to autopsy convention) demonstrating biventricular concentric hypertrophy. Note also patchy white discoloration of the myocardium indicating fibrosis, likely due to previous infarcts. No acute infarcts were appreciated macroscopically. (B) Aortic valve with fibrocalcific degeneration (arrows). (C) Mitral valve with fibrocalcific degeneration (arrows) (left atrium above, left ventricle below). Bar = 1 cm for all images.

**Figure 5:**
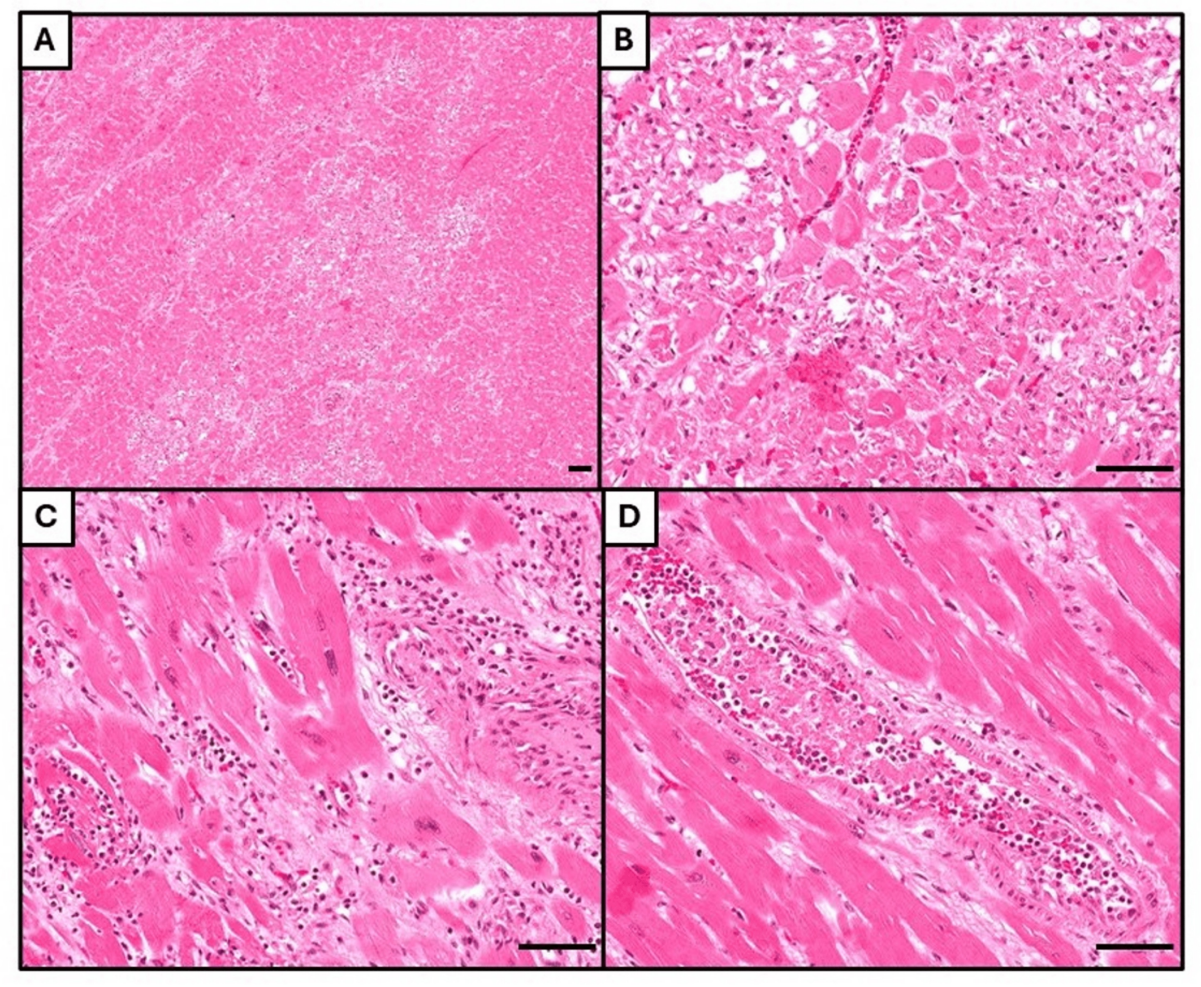
Microscopic examination of the heart. (A) Myocardium with multifocal infarcts. (B) Some infarcts are characterized by coagulative necrosis only, indicating an acute timeframe. (C) Other infarcts additionally have infiltration by neutrophils, indicating a subacute nature. (D) Myocardial vessel containing a microthrombus, with a granular appearance (top left) characteristic of platelet aggregation, and the presence of spindle-shaped fibroblasts (bottom right) showing evidence of organization. All histological images stained with hematoxylin & eosin, with bar = 50 μm.

**Figure 6:**
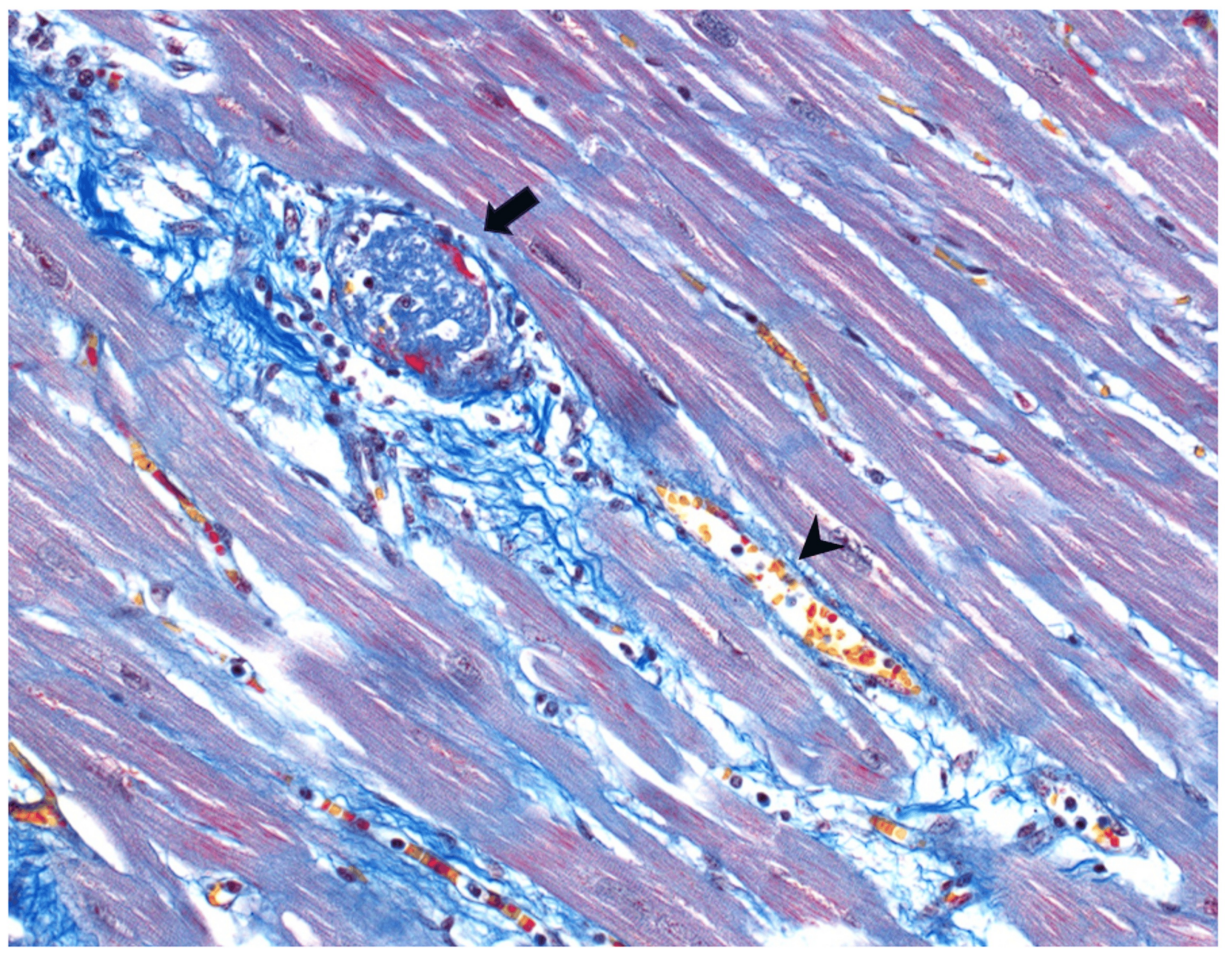
Martius Scarlet Blue (MSB) staining of the myocardium. Arrow: a well-formed thrombus (blue). Note the thin rim of fibrin (red) deposit at the edge of the thrombus. Arrowhead: a non-thrombosed blood vessel with red blood cells (RBC, yellow). Many small capillaries are also seen in the background with RBCs (yellow).

**Figure 7:**
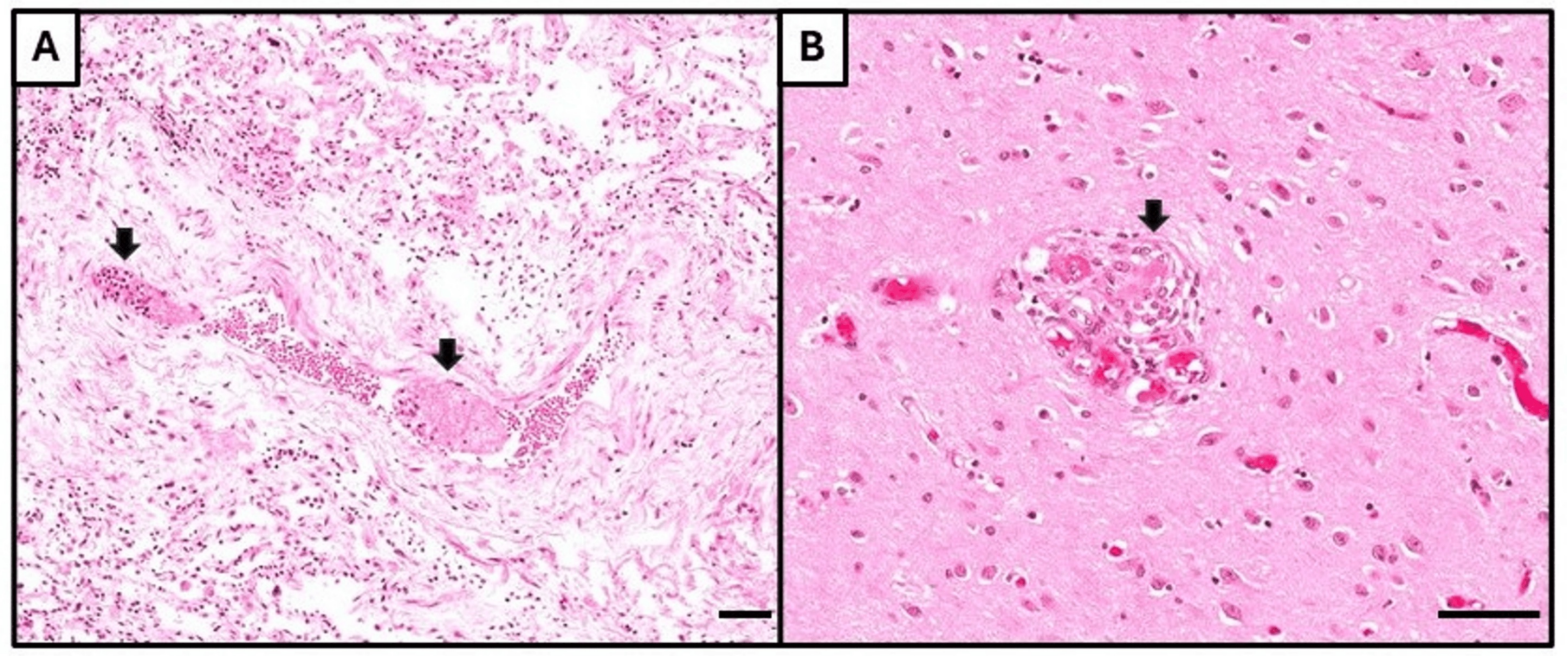
Microthrombi in other organs. (A) Pulmonary vessel containing two microthrombi (arrows), having a granular and branching texture indicative of platelet and fibrin aggregation. (B) Cerebral capillary containing possible microthrombus (arrow). All histological images stained with hematoxylin & eosin, with bar = 50 μm.

An autopsy was requested to elucidate the precise cause of death. On macroscopic examination, the deceased’s heart exhibited cardiomegaly (heart weight 444 g [reference range based on sex and body weight 182-390 g]), bilateral ventricular concentric hypertrophy, and severe fibrocalcific degeneration of the aortic and mitral valves, consistent with her history of mixed aortic stenosis-regurgitation (Figure 4) [2]. While there was no infarct appreciated macroscopically, microscopic examination of the myocardium revealed multifocal microinfarcts at varying ages-acute and subacute. This was associated with widespread microthrombi in the intramural vessels (Figures 5, 6). There were also focal pulmonary microthrombi without any gross pulmonary emboli (Figure 7A). In addition, there were rare possible thrombi in the cerebral capillaries of the brain (Figure 7B). There was no pulmonary or cerebral infarct identified.

In summary, the patient’s death was deemed to be caused by multifactorial cardiac decompensation, with the most important factor being multifocal microinfarcts, but with her valvular heart disease and a moderate degree of atherosclerosis as additional contributors.

## Discussion

APS is an autoimmune condition characterized by thrombotic events associated with circulating antiphospholipid antibodies (aPLs) including LA, anti-beta2-glycoprotein I (anti-B2GP1), and anti-cardiolipin antibodies (aCLs) [1]. This condition has an incidence rate of five per 100,000 people [1]. Long-term anti-coagulation with vitamin K antagonists (VKA) such as warfarin is the mainstay of treatment with target INR of 2.0 to 3.0 to prevent thrombotic events [3,4]. Close monitoring of INR is recommended for APS patients with history of thrombosis as subtherapeutic INR is associated with increased risk of recurrence [4]. CAPS is a very rare, aggressive variant of APS where patients can develop severe multiorgan thrombotic events that occur rapidly or simultaneously [1]. This condition affects approximately 1% of APS patients and is associated with an approximately 30-50% mortality rate [1]. The current criteria for a definite diagnosis of CAPS requires involvement of three or more organs/systems/tissues, histopathological confirmation of small vessel occlusion in at least one organ, two laboratory confirmations of aPL at least 12 weeks apart, and onset of symptoms within a week [1,3,5]. In this case, our patient presentation is consistent with definite CAPS given the history of APS diagnosis (positive LA and clinical manifestations of venous thromboembolism and recurrent pregnancy losses), and development of simultaneous organ system/tissue involvement including cardiac, cerebral, and pulmonary involvement with subsequent histopathological evidence of occlusion in all three organs on autopsy.

The pathophysiology of CAPS is multifactorial and complex, with aPLs triggering pro-inflammatory activity of endothelial cells and release of pro-inflammatory cytokines (ie. interleukin-6) that promote a pro-coagulant state and inhibit fibrinolytic pathways [1,6]. The complement system has been shown to be a major component of APS pathogenesis, with recent findings by Rand et al. demonstrating in vitro that pathogenic aPLs promote complement involved in neutrophil activation and increased prothrombotic cellular signaling [7,8]. Activation of neutrophils in APS promote release of neutrophil extracellular traps (NETs) which contribute to both arterial and venous thrombosis through platelet activation and thrombus formation [6]. Approximately 60% of CAPS cases have one or more identified trigger events, most often surgery, pregnancy, trauma, neoplasm, contraceptives, and cessation of anticoagulant therapy [1,3].

Our case highlights the challenges of managing cases of APS in patients presenting with life-threatening bleeding. Modification and cessation of anticoagulant therapy in APS patients have been suggested as precipitating factors for CAPS. Katikireddi and Kandiah reported three cases of CAPS following cessation of warfarin initially prescribed for thrombotic events as well as cessation of anticoagulant therapy during admission for surgery [9]. A study of two CAPS cases identified the introduction of rivaroxaban coinciding with CAPS onset when used as a bridging therapy for a prior anticoagulant [10]. Similarly, another study by Stammler et al. observed that of 61 patients treated with anticoagulants prior to CAPS onset, 32 had recently switched to a bridging therapy, begun a direct oral anticoagulant, or had subtherapeutic treatment (INR < 2) on VKAs such as warfarin [3]. Trauma and bleeding may be a trigger for CAPS as there is an increased risk of thrombosis possibly due to the inflammatory response [11]. Although there have been multiple cases in the literature on CAPS triggered from situations such as anticoagulant cessation, this case we present is unique as our patient died of CAPS as a consequence of management for another life-threatening medical condition. Our patient had presented with a GI bleed and required an EGD and required multiple transfusions of packed red blood cells and platelets upon admission. It is possible that a combination of these factors (cessation of treatment, GI bleed) contributed to this patient’s episode of CAPS, though there was no single definitive cause determined. The widespread microthrombi possibly contributed to a further persistent decline in hemoglobin and platelet levels, creating a vicious cycle. In such scenarios, management is dictated by the most immediate life-threatening complication, and modification of factors known to trigger CAPS such as cessation of warfarin therapy for management of a GI bleed highlights the difficulty in balancing bleeding and thrombotic complications with the risk of CAPS.

Catastrophic APS is characterized by a spectrum of clinical symptoms and can manifest as arterial and venous thrombosis in any organ. Multiorgan system failure is common, with a review of 571 CAPS episodes finding that the organs most affected were kidneys (74%), brain (56%), lungs (55%), and heart (53%) [5]. Cardiac involvement includes myocardial infarction, heart failure, and valve disease, while pulmonary involvement often manifests as acute respiratory distress syndrome or pulmonary embolism [1]. Rising cardiac biomarkers such as troponin serve as useful indicators of worsening cardiac injury in APS patients which may occur in the context of subtherapeutic INR levels [4,12]. In this case, there were clinical symptoms of both cardiac and respiratory organ involvement, developing supraventricular tachycardia with rising troponin as well as dyspnea with bilateral pleural effusions. Our patient’s history of cardiovascular disease (including diffuse myocardial microinfarcts, atherosclerosis, cardiomegaly, and valvular heart disease) along with widespread microthrombi in intramural vessels and increasing troponin levels possibly indicated worsening myocardial injury. Coinciding with a recent history of GI bleed, this likely would have contributed to the cardiac decompensation that occurred following onset of CAPS. Advanced age poses an additional challenge in terms of prognosis given older APS patients are more prone to have cardiovascular risk factors and have higher mortality rates with CAPS [13]. Current understanding of CAPS management in older patients is poor as the majority of documented CAPS patients are in women aged 11 to 60, with a median age of 37 [14].

The current standard of treatment of CAPS is an aggressive and timely triple therapy combination of anticoagulants, corticosteroids, and either therapeutic plasmapheresis or intravenous immunoglobulin, which is associated with a survival rate of 72% [1]. Clinical practice guidelines recommend treatment prior to diagnostic confirmation if there is reasonable suspicion especially given that certain criteria (such as aPL testing) are not appropriate in an acute setting [1,5]. Therefore, clinical expertise is required for an accurate and timely diagnosis. However, given the low prevalence of CAPS and the non-specific manifestations it can easily be missed in a differential. A similar case was reported by Lim et al. in a 70-year-old patient who presented with idiopathic CAPS manifesting in GI bleed, DVT, acute renal failure, and cardiac arrest despite being on therapeutic doses of warfarin [15]. This patient presentation was recognized and treated successfully with the standard triple combination therapy. Our case initially developed epigastric pain with a rise in troponin with no notable changes on EKG and echocardiogram followed by acute onset of dyspnea. Initial clinical suspicion included myocardial infarction, heart failure, pneumonia, pulmonary embolism, with presumed pre-renal AKI from volume contraction. Therefore, no CAPS-specific protocol was initiated during her stay in hospital and CAPS diagnosis was based on the multisystem tissue involvement (cardiac, pulmonary, and cerebral) identified on autopsy. It is likely that the non-specific constellation of symptoms resulting in inadequate treatment was consequential in her prognosis.

Emerging research on treatments for APS/CAPS provides a hopeful outlook for future cases. For primary prophylaxis, statins have been suggested to protect against thrombosis [16]. Daratumumab has been demonstrated to reduce aPL titers and treat APS patients that are refractory to first-line anticoagulant therapy, with the Daratumumab in Primary Antiphospholipid Syndrome (DARE-APS) trial launched in 2023 to study the use of daratumumab in primary APS patients [17,18]. NETs have been found to promote thrombus growth in APS patients and anti-neutrophil medications such as defibrotide were demonstrated in a patient case to induce remission of CAPS [19,20]. Rapid complement sequencing may serve a role in the future as an important biomarker in the diagnosis of CAPS and inhibition of complement-driven inflammation may be a potential target for treatment however both remain areas of active research [7]. Development of a rapid, CAPS-specific diagnostic test will provide physicians with clarity and enable timely, appropriate treatment for more complex cases such as the one presented here, where CAPS may be difficult to distinguish clinically from other life-threatening conditions. Additionally, incorporation of possible adjunct thrombolytic therapies in the management of APS may help prevent long-term complications and improve prognosis of CAPS.

## Conclusions

In summary, we report a case of CAPS that occurred in a patient undergoing treatment for severe anemia in the context of GI bleed. Given the patient’s non-specific respiratory and cardiovascular signs and symptoms, CAPS was not recognized, and she was treated unsuccessfully with only supportive therapy and blood transfusions. This case highlights the difficulty in an accurate and timely diagnosis of CAPS, especially in the context of another life-threatening condition. Additionally, our case demonstrates the complexity in balancing the risk of CAPS in APS patients when managing another serious health issue such as bleeding. In patient care, appropriate clinical suspicion and acumen are important in identifying and treating life-threatening syndromes such as CAPS.
